# What is dry? Exploring metabolism and molecular mobility at extremely low water contents

**DOI:** 10.1093/jxb/eraa579

**Published:** 2021-02-27

**Authors:** Jill M Farrant, Henk W M Hilhorst

**Affiliations:** 1 Department of Molecular and Cell Biology, University of Cape Town, Cape Town, South Africa; 2 Laboratory of Plant Physiology, Wageningen University and Research, Wageningen, The Netherlands

**Keywords:** Desiccation tolerance, glassy state, liquid-liquid phase separations, longevity, molecular mobility, Natural Deep Eutectic Solvents (NaDES)

## Abstract

This article comments on:

**Candotto Carniel FC, Fernandez-Marín B, Arc E, Craighero T, Laza MJ, Incerti G, Tretiach M, Kranner I**. 2021. How dry is dry? Molecular mobility in relation to thallus water content in a lichen. Journal of Experimental Botany **72**, 1576–1588

**Desiccation tolerance is defined as ‘the ability of tissues to survive loss of 95% of cellular water or dehydration to tissue water contents of ≤0.1 g H_2_O g^–1^ dry mass (DM)’. This trait is common in reproductive structures such as seeds, but relatively rare in vegetative tissues, occurring in only 330 (0.86%) species of vascular plants (** Box 1) (Proctor and Pence, 2002**). Among non-tracheophytes, it is present in virtually all lichens (Kranner *et al.*, 2009) and in 210 (1.04%) bryophytes (Wood, 2007). The ability to survive such extreme water loss has invoked much scientific interest which, among other things, has facilitated applications in germplasm conservation via seed storage (Bewley *et al.*, 2013) and, in the case of vegetative desiccation tolerance, towards production of extremely drought-tolerant crops for food security in a hotter dryer future (Hilhorst and Farrant, 2018).**

Box 1. **The evolution of vegetative desiccation tolerance**

Vegetative desiccation tolerance (DT) is thought to have its origin in the colonization of the land some 500 million years ago where survival strategies were aiming at either minimizing the loss of water or tolerating it. During dry periods, the earliest land plants were probably in equilibrium with the environment and, hence, must have been desiccation tolerant if the dry periods were long enough. With the occurrence of specialization of plant functions, such as the vascular systems in the Tracheophytes, as well as occupation of different niches, the requirement for full vegetative DT diminished and was diverted to seeds and pollen. Current consensus is that extant vegetative DT was rewired from reproductive DT in at least 13 independent evolutionary events. However, a loss of DT function during the course of evolution may also be considered. The former hypothesis may explain the striking molecular similarities between seed and vegetative DT mechanisms whereas the latter assumes the permanence of reproductive DT after the loss of vegetative DT.

Of particular interest is the observation that metabolic activity in terms of transcription, translation, and metabolism can occur at relative water contents (RWCs) below 30% (~0.7 g H_2_O g^–1^ DM; species and study dependent), with respiration (in many instances) ceasing only at 10% RWC (0.1 g H_2_O g^–1^ DM) (reviewed in Costa *et al.*, 2017; Farrant *et al.*, 2017; Banchi *et al.*, 2018; Zhang and Bartels, 2018; Oliver *et al.*, 2020). This implies that molecular mobility is possible even at extremely low water contents. This observation has been ably demonstrated in the desiccation-tolerant lichen *Flavoparmelia caperata*, where Candotta Carniel *et al.* (2021) have shown that enzyme activity occurs at 0.17 g H_2_O g^–1^ DM (10% of initial water content), ceasing only at between 0.12 g H_2_O g^–1^ DW and 0.08 g H_2_O g^–1^ DW. Use of dynamic mechanical thermal analysis (DMTA) led the authors to propose that the cytoplasm is in an amorphous ‘rubbery’ state at 0.17 g H_2_O g^–1^ DM (in which the cytoplasmic viscosity is five times higher than in the liquid state) but that it enters an amorphous glassy state at 0.03 g H_2_O g^–1^ DM, at which point no metabolic activity was recorded.

Key questions concern how this is possible and what are the consequences. Most studies on the nature of the dry state have used seeds, with a particular emphasis on understanding seed longevity, namely the ability to survive long-term dry storage, for example in seed banks. Such studies have concentrated predominantly on tissues in the desiccated state (at or below 0.1 g H_2_O g^–1^ DM). Theoretically, no chemical or enzymatic reactions can occur in this glassy state, other than solid state oxidation, peroxidation, and carbonylation reactions of molecules in close proximity—in the solid cytosol (Ballesteros *et al.*, 2020). Yet, significant changes may still occur in this dry state, including seed after-ripening which releases dormancy (Bewley *et al.*, 2013), transcript levels (Cadman *et al.*, 2006), and enzyme activity (Leubner-Metzger, 2005). This has led to the conclusion that the cells in dry seeds and, for that matter, those of dry desiccation-tolerant vegetative tissues, may contain ‘islands’ of mobility in the absence of free or weakly bound water. Examples are oil droplets which remain fluid within dry cells, potentially allowing longer range diffusion of small molecules such as reactive oxygen species (ROS) and, hence, chemical reactions (Ballesteros *et al.*, 2020). Additionally, differences in the concentrations of cellular compounds created by drying of a heterogeneous cell may provide a localized liquid environment in the absence of detectable water, for example by pockets of natural deep eutectic solvents (NaDES). While the presence of NaDES in desiccation-tolerant systems has been proposed (duToit *et al.*, 2020; Oliver *et al.*, 2020), they are not easily demonstrated. Furthermore, the biochemically inhomogeneous nature of the subcellular environment in desiccated tissues may result in fragile glasses, which may change abruptly from a solid to a fluid phase over a narrow temperature range, potentially allowing localized molecular mobility (Ballesteros and Walters, 2019). Another possibility of generating localized mobility in dehydrating cells was recently suggested for the desiccation-tolerant brine shrimp *Artemia franciscana*. Here, liquid–liquid phase transitions were demonstrated. These were enabled by the presence of a group 6 late embryogenesis abundant (LEA) protein, which is present only in desiccation-tolerant seeds and in the vegetative tissues of desiccation-tolerant angiosperms. Domains in this protein drive the formation of protein condensates that act as protective compartments for desiccation-sensitive proteins and potentially allow molecular interactions, such as protein binding (Belott *et al.*, 2020).

To date, only two other studies have reported on the use of biophysical tools such as DMTA to couple observations of subcellular mobility with enzyme activity in desiccation-tolerant vegetative tissues, both utilizing the xanthophyll cycle enzymes as a proxy for metabolic activity (Fernández-Marín *et al.*, 2013, 2018). Due to the general accumulation of glass-forming sugars (sucrose and oligosaccharides) during dehydration in desiccation-tolerant vegetative tissues, and the presence of numerous LEA proteins in higher plants, it has been commonly assumed that such glasses exist in the desiccated state (Zhang *et al.*, 2016; Farrant *et al.*, 2017). However, it is highly likely that there are differences in the ultimate chemo-physical nature of subcellular environments between tissues such as seeds and leaves, with variations across species. We propose that this in turn is related to the nature of the environment (niche) and the function of the tissue (reproductive, e.g. seed or vegetative propagation)

As illustrated in Box 2, in dry seeds, a large proportion of the subcellular environment is occupied by starch-containing plastids, protein and/or lipid bodies, with a considerably reduced cytoplasmic area, in which sugar glasses occur. In vegetative tissues, mechanical stabilization in the dry state is achieved in several ways in different species, by a combination of increased vacuolation (such vacuoles are proposed to contain ‘compatible solutes’, the nature of which is likely to vary between species) and by wall folding, features that usually occur in inverse proportions (Farrant *et al.*, 2017; Oliver *et al.*, 2020). Regardless of the mechanism involved, considerably larger volumes of cytoplasm are produced that require stabilization and vitrification. In a review of metabolites accumulated in desiccating leaves of angiosperms, du Toit *et al.* (2020) point out that neither the absolute metabolite concentrations nor the proportions of sugar-forming glasses account for sugar glasses as the sole mechanism of subcellular stabilization. Organic acids (citrate and malate in particular) and some amino acids, which are all prone to NaDES formation, exist in much higher amounts than sucrose. du Toit *et al.* (2020) propose that accumulation of NaDES-forming metabolites that are possibly tailored to each organelle or cytoplasmic location enables localized pockets of ongoing metabolism at extremely low water contents. The formation of a citrate–sucrose NaDES in mitochondria has been proposed to facilitate respiratory activity observed at RWCs as low as 10% in *Xerophyta schlechteri* (Radermacher *et al.*, 2019) and, indeed, *in vitro* studies have shown that the mitochondrial antioxidant enzyme glutathione reductase (GR) has increased efficiency in such NaDES, indirectly confirming the suggestion that NaDES enable mitochondrial functions and antioxidant activities at low water contents (du Toit *et al.*, 2020). This could explain the observed enzymatic activities of the xanthophyll cycle enzymes in the thalli of *F. caperata* at 10% water content and also account for the ‘rubbery’ state of the tissues at this water content.

Box 2. **Schematic representation of subcellular organization in desiccation-tolerant tissues at 10% relative water content.**

The presence of NaDES and glass-forming matrices is indicated by colour shading, and the presence of LEA proteins and liquid droplets is indicated.

(A and B) Cells typical of lipid- and starch-rich seeds, respectively. (C) Typical subcellular organization in leaves of poikilochlorophyllous angiosperms, in which chlorophyll is degraded and thylakoids are dismantled in dry tissues. As typified in desiccation-tolerant *Xerophyta* species, such species tend to have numerous small vacuoles containing potentially NaDES-forming compatible solutes and have relatively rigid cell walls, in this regard being ‘seed like’. (D) Cellular organization of photosynthetic tissues of homoiochlorophyllous species, which retain and protect the photosynthetic apparatus during desiccation. Homoiochlorophylly is an evolutionarily ancient strategy, being present in all non-vascular clades, eudicots, and most C_4_ monocots. Mechanical stabilization is achieved by wall flexibility and some degree of vacuolation, the extent of which varies among species.

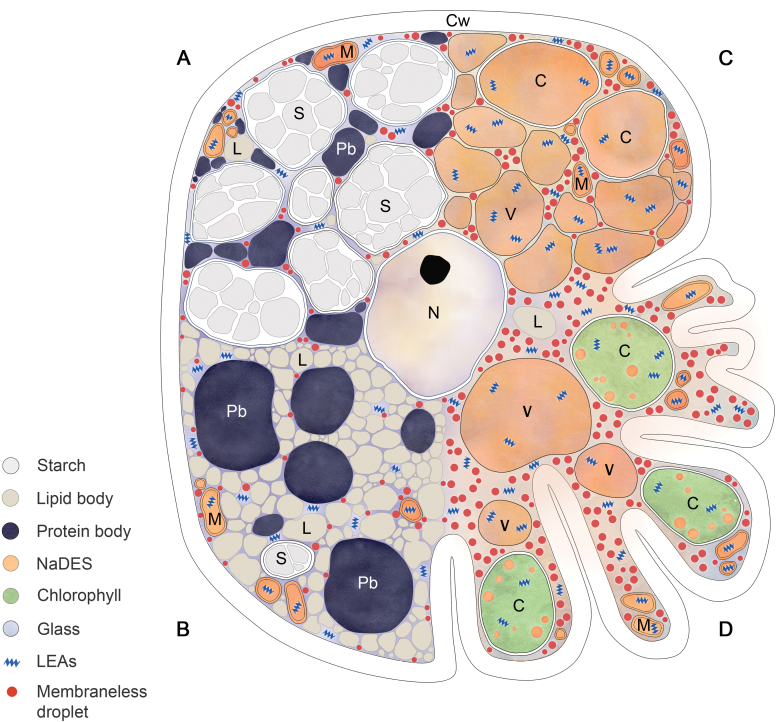

The nature of the degree of subcellular stabilization realized is highly likely to be related to organ or tissue function (e.g. seed or vegetative tissues) and the environmental niche. Because of their relatively small size, most dry seeds are permanently in equilibrium with the surrounding air and they are able to remain viable for many years because of the presence of complex reserves and the nature of formed glasses. In contrast, the vegetative tissues of higher plants are unlikely to be in equilibrium with the surrounding air for long periods, because they occur in specific niches, with periods in the desiccated state lasting for only weeks to months. Thus, an investment in complex reserves and ultrastable glasses is perhaps not such a high priority as it is in seeds. Rather, a versatile system of localized NaDES and glasses could exist, giving cells a mechanical buffering capacity and enabling a certain degree of regulation of metabolism at extremely low water contents, allowing rapid recovery of metabolic activity (minutes to hours) that is observed in vegetative tissues.

In conclusion, desiccation tolerance is an extreme strategy, evolved for survival of prolonged periods without water. The success of this strategy relies on biochemical protection, involving many of the putative molecules that not only enable metabolism to occur even at very low water contents, but also allow the ultimate chemo-physical stabilization of the subcellular milieu in the desiccated state. The exact nature of the metabolites accumulated (albeit with many common candidates) varies between organs (seeds or leaves) and species. We propose that this is likely to be related to the longevity requirements of the dry state and the metabolic cost of the implementation of the biochemical protection strategy.

## Abbreviations:

C: chloroplast
Cw: ce
l: l wall
L: lipid
M: mitochondrion
N: nucleus
Pb: protein body
S: starch
V: vacuole.
